# New and Stale Bread

**Published:** 1881-02

**Authors:** 


### NEW AND STALE BREAD.

The nature of the difference between new and stale bread is far from being known. It is only lately that the celebrated French chemist, Boussingault, instituted an inquiry into it, from which it results that the difference is not the consequence of dessication, but solely of the cooling of the bread. If we take fresh bread into the cellar, or in any place where it cannot dry, the inner part of the loaf, it is true, is found to be crumbly, but the crust is no longer brittle. If stale bread is taken into the oven again, it assumes all the qualities of fresh baked bread, although in the hot oven it must undoubtedly have lost part of its moisture. M. Boussingault has made a fresh loaf of bread the subject of minute investigation, and the results are interesting. New bread, in its smallest parts, is so soft, clammy, flexible and glutinous, (in consequence of the starch during the process of fermenting and baking being changed into mucilaginous dextrine), that by mastication it is with greater difficulty separated and reduced to smaller parts, and is less under the influence of the saliva and digestive juices. It consequently forms itself into hard balls by careless and hasty mastication and deglutition, becomes coated over by saliva and slime, and in this state enters the stomach. The gastric juice being unable to penetrate such hard masses, and being scarcely able even to act upon the surface of them, they frequently remain in the stomach unchanged, and, like foreign bodies, irritate and incommode it, inducing every species of suffering—oppression of the stomach, pain in the chest, disturbed circulation of the blood, congestion and pain in the head, irritation of the brain, and inflammation, apoplectic attacks, cramp and delirium.

After the Physicians.—The New York Legislature has passed a law requiring the registration of all practicing physicians. This law also compels a practitioner of medicines coming from another State to prove his standing before the faculty of a New York medical college. Physicians in good standing do not complain of this law. In fact, they did a good deal to get it passed, as a protection to the profession against empiricism. Human life is too valuable to be trifled with, and no means can be too rigid which are devised for its protection. In this view the New York law will meet with general approval.

